# Lattice Distortion and Low-Frequency Anharmonic Phonons Suppress Charge Recombination in Lead Halide Perovskites upon Pseudohalide Doping: Time-Domain Ab Initio Analysis

**DOI:** 10.1021/acs.jpclett.3c02850

**Published:** 2023-11-21

**Authors:** Teng-Fei Lu, Weibin Chu, Sraddha Agrawal, Zhihua Zhang, Oleg V. Prezhdo

**Affiliations:** † School of Materials Science and Engineering, Dalian Jiaotong University, Dalian 116028, Liaoning, China; ‡ Key Laboratory of Computational Physical Sciences (Ministry of Education), Institute of Computational Physical Sciences, Fudan University, Shanghai 200433, China; § Department of Chemistry, University of Southern California, Los Angeles, California 90089, United States

## Abstract

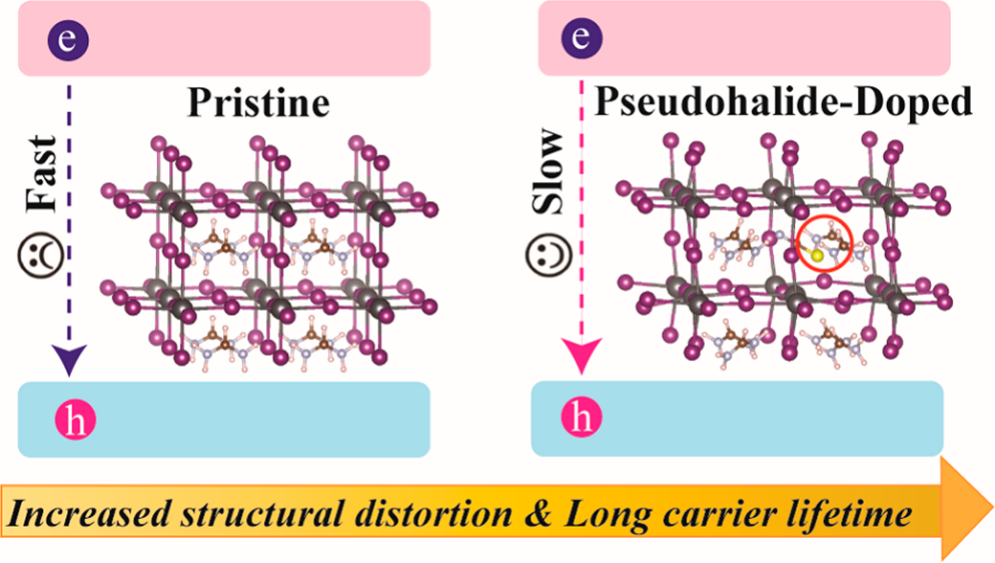

Perovskite solar cells have witnessed a surge in interest as a promising technology for low-cost, high-efficiency photovoltaics with certified power conversion efficiencies beyond 25%. However, their commercial development is hindered by poor stability and nonradiative losses that restrict their approach to the theoretical efficiency limit. Using ab initio nonadiabatic molecular dynamics, we demonstrate that nonradiative charge recombination is suppressed when the iodide in formamidinium lead iodide (FAPbI_3_) is partially replaced with pseudohalide anions (SCN^–^, BF_4_^–^, and PF_6_^–^). The replacement breaks the symmetry of the system and creates local structural distortion and dynamic disorder, decreasing electron–hole overlap and nonadiabatic electron–vibrational coupling. The charge carrier lifetime is found to increase with increased structural distortion and is the longest for PF_6_^–^. This work is fundamentally relevant to the design of high-performance perovskite materials for optoelectronic applications.

Organic–inorganic hybrid perovskites (OIHPs) have emerged as one of the most promising semiconducting materials in the realm of photovoltaic research, owing to their outstanding optoelectronic properties, including high absorption coefficients, long charge carrier diffusion lengths, and ambipolar charge transport.^1−4^ These fine features render the applications of such perovskites to extend beyond solar energy harvesting into light-emitting diodes, lasers, transistors and detectors, etc.^5−12^ The power conversion efficiency (PCE) of OIHPs has witnessed a staggering increase since the first report of a methyl ammonium halide cell in 2009,^13^ from an initial value of 3.8%^13^ to 25.5%,^14^ which is comparable to that of conventional silicon-based solar cells. Despite the impressive progress in the performance, the currently achievable efficiency of these perovskite solar cells (PSCs) is still well below the theoretical Shockley–Queisser limit of 33%^15^ for a single-junction device, suggesting the presence of additional channels of energy losses other than radiative pathways. Often, the nonradiative recombination of charge carriers has been identified as the predominant pathway that limits device performance. This entails an urgent need to understand the underlying mechanisms of nonradiative recombination losses and the relationship between the perovskite structure and charge carrier dynamics at the atomistic level to further push the efficiency of PSCs toward the thermodynamic limit.

Three-dimensional (3D) perovskites have a general chemical formula of ABX_3_, where A stands for a monovalent cation (MA^+^ = CH_3_NH_3_^+^, FA^+^ = CH(NH_2_)_2_^+^, or Cs^+^), specifically an organic cation for OIHPs, B stands for a divalent metal cation (Sn^2+^ or Pb^2+^), and X is a halide anion (Cl^–^, Br^–^, or I^–^).^16^ Among OIHPs, methylammonium lead iodide (MAPbI_3_) is the most widely studied photovoltaic material. However, it is intrinsically thermally unstable due to its low formation energy.^17^ Formamidinium lead iodide (FAPbI_3_) is generally preferred over MAPbI_3_ because of its superior thermal stability and charge carrier transport properties.^18,19^ Unfortunately, practical applications of FAPbI_3_ are seriously limited by the spontaneous transformation from its photovoltaically active phase to the unwanted but more stable phase.^20^ Long-term stability under ambient photo and thermal stresses remains a critical bottleneck for the large-scale commercialization of PSCs.^21^ This challenge has motivated efforts to improve device stability, and significant advancements in this direction are being made by developing novel perovskite materials. Composition engineering, especially using mixed cations and halide anions, has proven to be a feasible and effective approach to achieve stable, high-performance PSCs.^22,23^ In particular, mixtures of MAPbI_3_ with FAPbI_3_ have been demonstrated to stabilize the active phase and suppress ion migration, achieving excellent transport characteristics.^24^ Calculations show that alkali metal dopants greatly improve perovskite performance by passivating interstitial defects, therefore extending carrier lifetimes.^25^ Compared to the amount of effort that has been put into building perovskites based on mixed cations and halides, not much focus has been placed on using molecular anions as alternatives to halogen anions to modulate the PCE.

Pseudohalides, which are polyatomic analogues of halides such as thiocyanate (SCN^–^), tetrafluoroborate (BF_4_^–^), and hexafluorophosphate (PF_6_^–^), can serve as alternatives for X-site halides in perovskites because their ionic radii and chemical properties are similar to those of true halides.^26^ A wider range of negatively charged anions (pseudohalides) may form the ABX_3_ stoichiometry with Pb^2+^. Zhang et al. reported that introducing the BF_4_^–^ anion in a mixed-ion perovskite crystal frame resulted in a slight lattice relaxation, a longer photoluminescence lifetime, and improved charge transport in the perovskite solar cell.^27^ By utilizing relativistic electronic structure calculations, Hendon and co-authors demonstrated that substitution with BF_4_^–^ and PF_6_^–^ anions in hybrid perovskites can form wide bandgap dielectric compounds.^28^ Tai et al. reported that the incorporation of the SCN^–^ anion as a dopant improved the moisture resistance and photovoltaic performance of PSC devices.^29^ Given these interesting findings, a systematic study on the detailed mechanism of PCE improvement with pseudohalides in OIHPs is needed. This can be achieved by an atomistic investigation of the structural and electronic properties, electron–vibrational interactions, and charge carrier dynamics.

Motivated by experimental studies, we report here an ab initio time domain investigation of the substitution by the pseudohalide anions PF_6_^–^, BF_4_^–^, and SCN^–^ as “dopants” in FAPbI_3_ and provide a mechanistic understanding of the nonradiative electron–hole recombination and reduced charge and energy losses. We demonstrate that nonradiative charge recombination is suppressed in FAPbI_3_ doped with pseudohalide anions due to changes in the properties of the inorganic sublattice. Minor changes in the PbI_3_^–^ octahedra can affect the electronic properties of the perovskite to a significant extent. The anion doping expands the inorganic sublattice, distorts the PbI_3_^–^ octahedra, and creates structural asymmetries, which in turn change the electronic properties of the sublattice. Dynamically, the anion substitution activates low-frequency lattice vibrations that are responsible for transient disorder and decreased electron–hole interactions. The simulations illustrate that the synergistic effect of static and dynamic lattice disorder decreases NA coupling, thereby slowing the nonradiative electron–hole recombination in polyanion-substituted FAPbI_3_. The atomistic details of the mechanism of the nonradiative charge and energy losses provide guidelines for further improvement of the performance of hybrid perovskites.

To simulate the nonradiative recombination processes in the FAPbI_3_ perovskite with different pseudohalide dopants, we performed ab initio nonadiabatic molecular dynamics (NAMD) simulations with real-time time-dependent density functional theory (TD-DFT) in the Kohn–Sham representation.^30−32^ The lighter electrons were treated quantum-mechanically, whereas the heavier atoms were described semiclassically. The charge recombination dynamics were investigated using the decoherence-induced surface hopping (DISH) technique, which includes the loss of coherence within the electronic system due to coupling to quantum phonons.^33,34^ The decoherence time is estimated as the pure dephasing time using the optical response theory.^35,36^ In order to further reduce the computational cost, classical path approximation (CPA) was used,^37^ in which the atomic dynamics were assumed to be weakly dependent on the quantum state of the electronic subsystem, as compared to thermal atomic fluctuations. This methodology has already been widely applied to study excited-state dynamics in a broad range of systems, including perovskites.^38−59^

The ground-state geometry optimization, electronic structure calculations, and room-temperature MD were performed with the Vienna Ab Initio Simulation Package (VASP).^60^ The Perdew–Burke–Ernzerhof (PBE) exchange-correlation functional was adopted.^61,62^ The van der Waals interactions were described by the Grimme DFT-D3 method.^63^ Both the structural optimization and the adiabatic MD employed a 4 × 4 × 4 Γ-centered Monkhorst–Pack *k*-mesh grid.^64^ The NA couplings (NACs) were computed for the Γ-point, as the structures had direct bandgaps located at the Γ-point. The plane wave energy cutoff was 400 eV. The geometry optimization at 0 K was stopped when the Hellmann–Feynman forces on each atom were smaller than 0.01 eV/Å. Then, the systems were heated at 300 K through repeated velocity rescaling and further equilibrated for 6 ps with a 1 fs atomic time step in the NVE ensemble. Next, 500 initial conditions were selected randomly from the last 4 ps of the MD trajectories, and NAMD simulations were carried out using 1000 random number sequences to sample the surface hopping probabilities for each initial geometry. The NAMD simulations were performed with the Python eXtension for Ab Initio Dynamics (PYXAID) code.^33,37^

To investigate the electron–hole recombination dynamics in different pseudohalide anion-substituted perovskites, we first constructed a 96-atom (2 × 2 × 2) simulation supercell of the FAPbI_3_ cubic phase and generated pseudohalide-mixed perovskite structures through partial halide substitution. Figure 1 shows the optimized stoichiometric structure for pristine FAPbI_3_ (Figure 1a) and the substituted structures (Figures 1b–d), in which one of the I^–^ ions has been replaced by BF_4_^–^, PF_6_^–^, or SCN^–^, respectively. The calculated averaged Pb–I bond length in the optimized pristine FAPbI_3_ was 3.174 Å, which is consistent with the experimental (3.181 Å) and theoretical (3.177 Å) data.^65^ After the pseudohalide anion substitution, the inorganic Pb–I sublattice around the doping site expands and the Pb–Pb distances increase (Table 1). Thus, the distances between the key Pb atoms, marked in Figure 1a, are 6.57, 6.98, and 7.26 Å for Pb_1_–Pb_2_ and 6.51, 6.56, and 6.61 Å for Pb_2_–Pb_3_ in I_SCN_, I_BF_4__ and I_PF_6__, respectively, as compared to 6.35 Å for Pb_1_–Pb_2_ and 6.50 Å for Pb_2_–Pb_3_ in FAPbI_3_. Structural fluctuations at room temperature led to a slight decrease in the canonically averaged Pb–Pb distances relative to the 0 K data; however, the dopant-induced expansion trend remained (Table 1).

**Figure 1:**
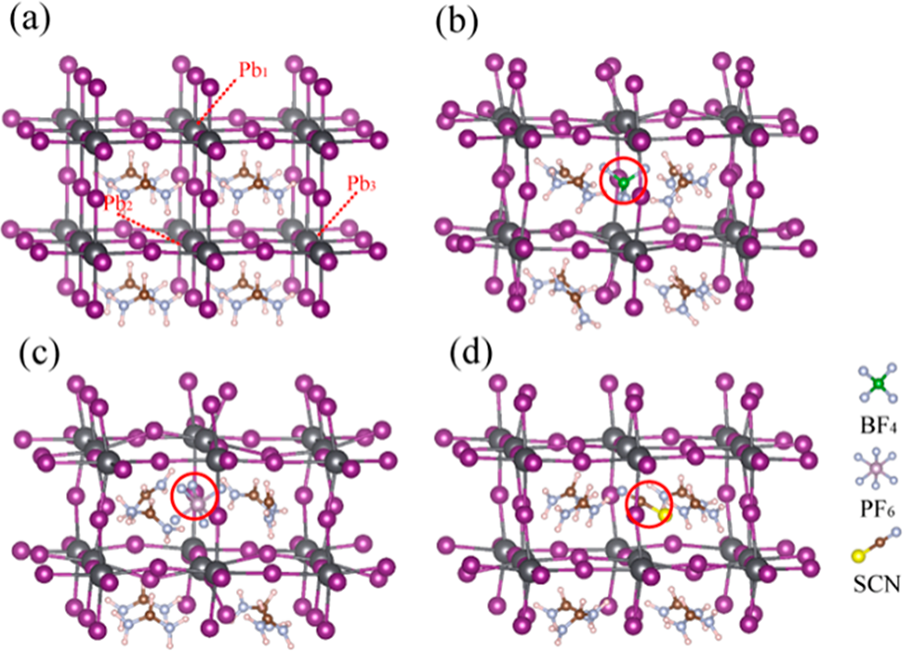
Structures of (a) pristine FAPbI_3_, (b) FAPbI_3_ with I_BF_4__, (c) FAPbI_3_ with I_PF_6__, and (d) FAPbI_3_ with I_SCN_. The red circles indicate the sites where iodine atoms are substituted by the respective pseudohalide anions.

**Table 1:** Pb–Pb Distances ^a^Pb_1_–Pb_2_ and ^a^Pb_2_–Pb_3_ at 0 K and the Averaged Pb–Pb Distances ^b^Pb_1_–Pb_2_ and ^b^Pb_2_–Pb_3_ at 300 K around the Anion-Substituted Site (See Figure 1)

	^a^Pb_1_–Pb_2_ (Å)	^a^Pb_2_–Pb_3_ (Å)	^b^Pb_1_–Pb_2_ (Å)	^b^Pb_2_–Pb_3_ (Å)
FAPbI_3_	6.35	6.50	6.32	6.46
I_BF_4__	6.98	6.56	6.80	6.52
I_PF_6__	7.26	6.61	7.16	6.60
I_SCN_	6.57	6.51	6.52	6.49

The PF_6_^–^ substitution showed the maximum increase in the Pb–Pb distances and the highest local lattice expansion among all of the systems. Distortion of the octahedral structure is known in mixed-ion perovskites,^66,67^ leading to lattice strain that balances different-sized cations/anions. The molecular orbitals of pseudohalides such as BF_4_^–^ (tetrahedral shape) can weakly hybridize with the atomic orbitals of Pb^2+^ compared to iodide (spherical shape), and hydrogen bonds can form between pseudohalides and FA^+^. Such additional chemical bonding contributes to the improved chemical stability of the doped perovskites. The local expansion of the inorganic Pb–I sublattice modulates the electronic properties and electron–vibrational coupling, which in turn influences the electron–hole recombination.

The projected density of states (PDOS) of pristine FAPbI_3_ and the doped systems I_PF_6__, I_BF_4__, and I_SCN_ are shown in Figure 2. The PDOS is separated into the FA, Pb, I, and pseudohalide contributions. The conduction band minimum (CBM) and valence band maximum (VBM) of perovskites are formed primarily by the Pb-6p and I-5p/Pb-6s atomic orbitals, respectively. The electronic states near the Fermi level are supported by the Pb and I atoms; therefore, the PbI_6_ octahedra determine the relevant electronic properties. Among all of the pseudohalide atoms, only the SCN^–^ group makes a contribution to the valence band, although it is almost negligible. The pseudohalide anions do not contribute to the band edge states, and hence, they have no direct influence on the electron–hole recombination. They instead influence the recombination in an indirect way: by perturbing the structure and motions of the inorganic Pb–I framework.

**Figure 2:**
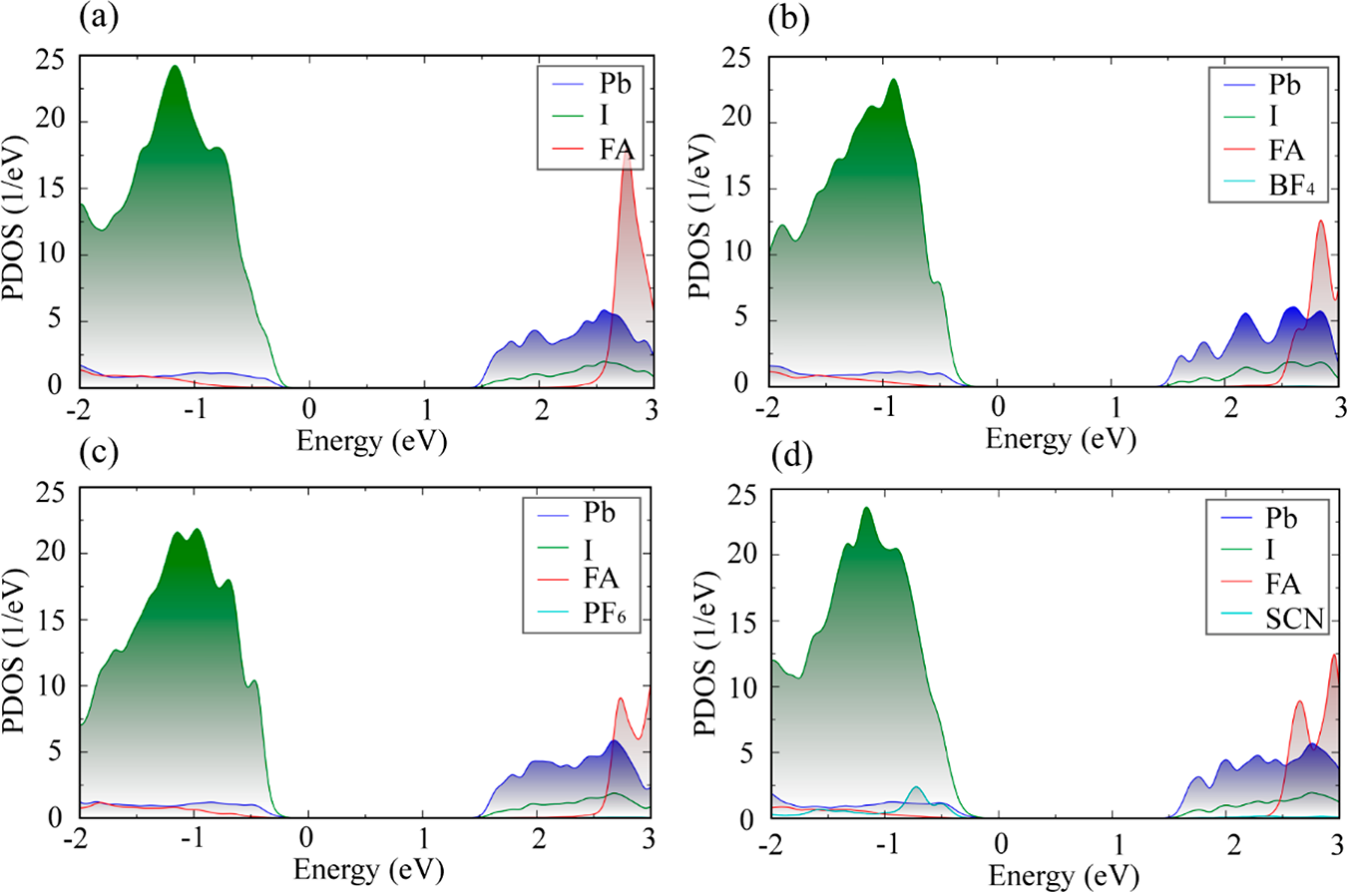
Atom-projected DOS for (a) pristine FAPbI_3_ and the doped systems (b) I_BF_4__, (c) I_PF_6__, and (d) I_SCN_ at 300 K. The energy reference is given at the Fermi level.

The calculated canonically averaged direct bandgap of pristine FAPbI_3_ was 1.80 eV, (Table 2), which is in agreement with previous DFT calculations.^68^ The canonically averaged bandgaps in the anion-substituted systems ranged from 1.82 to 1.86 eV, which are again consistent with the experimental trend.^27^ No additional electronic levels (traps) were introduced in the bandgap for the doped systems.

**Table 2:** Canonically Averaged Bandgap, Absolute NA Coupling, Pure Dephasing Time, and Nonradiative Electron–Hole Recombination Time

	average bandgap (eV)	NA coupling (meV)	dephasing (fs)	recombination (ns)
FAPbI_3_	1.80	0.426	9.03	6.36
I_BF_4__	1.82	0.389	8.49	12.56
I_PF_6__	1.86	0.353	9.06	15.62
I_SCN_	1.83	0.399	10.33	8.63

The electron–hole recombination is determined primarily by electron–vibrational NA coupling (NAC). The NAC, *d*_*jk*_, is calculated as the overlap between the electronic states *j* and *k* at sequential time steps:^69,70^
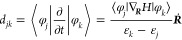
1where *H* is the electronic Hamiltonian, φ_*j*_, φ_*k*_, *ε*_*k*_, and *ε*_*j*_ are the wave functions and energies of the electronic states *k* and *j*, and ***Ṙ*** is the atomic velocity vector. The last expression demonstrates that the NAC is inversely proportional to the energy difference *ε*_*k*_ – *ε*_*j*_, grows with the atomic velocity ***Ṙ*** (and hence temperature), and depends on the electron–vibrational coupling matrix element ⟨φ_*j*_|∇_*R*_*H*|φ_*k*_⟩. The latter depends on the relative localization of the two wave functions, which are sensitive to composition and thermal disorder.^71−73^ The CBM and VBM charge densities, which characterize the localization of electrons and holes, are shown in Figure 3. Figure 3a demonstrates that in pristine FAPbI_3_, the VBM is localized primarily on I atoms, while the CBM is distributed over Pb atoms, which is consistent with the PDOS analysis (Figure 2). The localization of the CBM and the VBM on different atoms is beneficial for achieving a decreased wave function overlap and a small NAC. The anion substitution influences the localization of both the VBM and the CBM. Because the VBM is supported by iodides, by removing an iodide, the anions also remove the hole density from the corresponding spatial region. The CBM is influenced indirectly by the distortion of the Pb sublattice. Overall, the electron and hole wave functions become more localized upon pseudohalide doping. Provided that they are not being localized in the same place, their overlap should be reduced and the NAC decreased. Indeed, the NAC decreases in the following order (Table 2): FAPbI_3_ > I_SCN_ > I_BF_4__ > I_PF_6__, suggesting that the charge carrier lifetime should increase in this same order. The NAC trend correlates with the changes in the Pb–Pb distances (Table 1). The largest change in I_PF_6__ leads to the smallest NAC.

**Figure 3:**
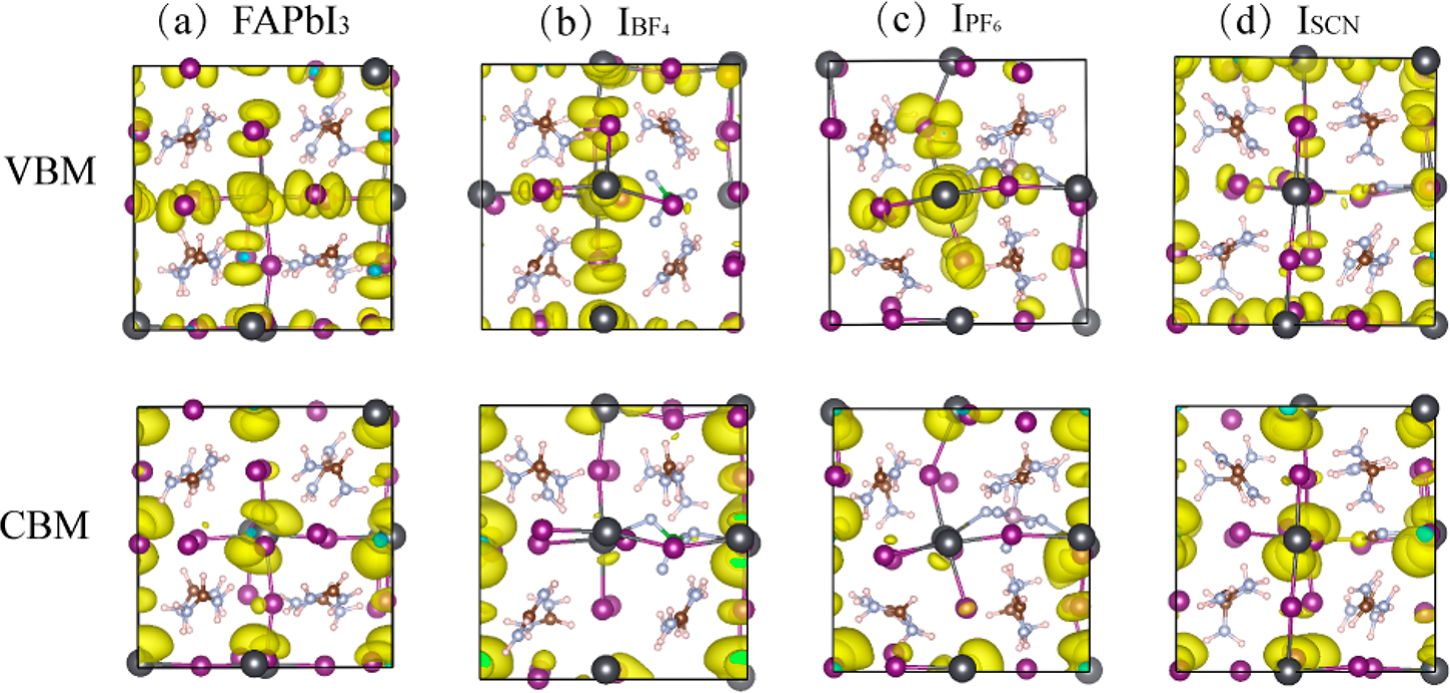
Orbital spatial charge densities of the VBM and CBM for the representative configuration at 300 K in (a) FAPbI_3_, (b) I_BF_4__, (c) I_PF_6__, and (d) I_SCN_.

Excess electronic energy is accommodated by phonons during nonradiative charge recombination. Electron–vibrational interactions generate elastic and inelastic scattering, both affecting the excited-state lifetime. Inelastic electron–phonon scattering leads to an energy exchange between the electronic and vibrational subsystems and is characterized by the NAC. Significant amounts of electronic energy are deposited into atomic degrees of freedom during nonradiative charge recombination. Elastic electron–phonon scattering destroys the coherence formed between the initial and final states. Because the formation of coherence is necessary for a quantum process to occur, fast decoherence can drastically slow down electron–hole recombination.^74,75^

In order to identify the vibrational motions that couple to the electronic transition across the bandgap, we computed Fourier transforms of autocorrelation functions (ACFs) of the bandgap fluctuations, δ*E*(*t*), from its canonically averaged values:
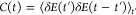
2The obtained spectral densities
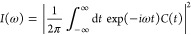
3are reported in Figure 4. Several modes in the 30–300 cm^–1^ frequency range couple to the electronic transition, including vibrations arising from the inorganic Pb–I framework, the organic FA cation, and the pseudohalide anions. The main high-intensity peaks that are observed at low frequencies (<100 cm^–1^) can be attributed to the bending and stretching modes of the slow Pb–I framework, which generates the NAC. The peaks at higher frequencies are much weaker in amplitude and arise from motions of the organic cations. FA and the pseudohalides contain light atoms and move much faster than the Pb–I framework; however, they do not contribute to the band edge states. Their influence on the electron–hole recombination is indirect, through coupling to the Pb–I lattice. The influence spectra shift to lower frequencies in the presence of the pseudohalide anions (Figure 4). The peak around 30 cm^–1^ is related to the octahedron distortion in the doped systems. Lower-frequency motions exhibit smaller atomic velocities (at a given temperature), hence reducing the NAC, which is proportional to the velocity (eq 1. Furthermore, low-frequency motions create dynamic disorder, which partially localizes the electron and hole wave functions and reduces the NAC as well.^76,77^

**Figure 4:**
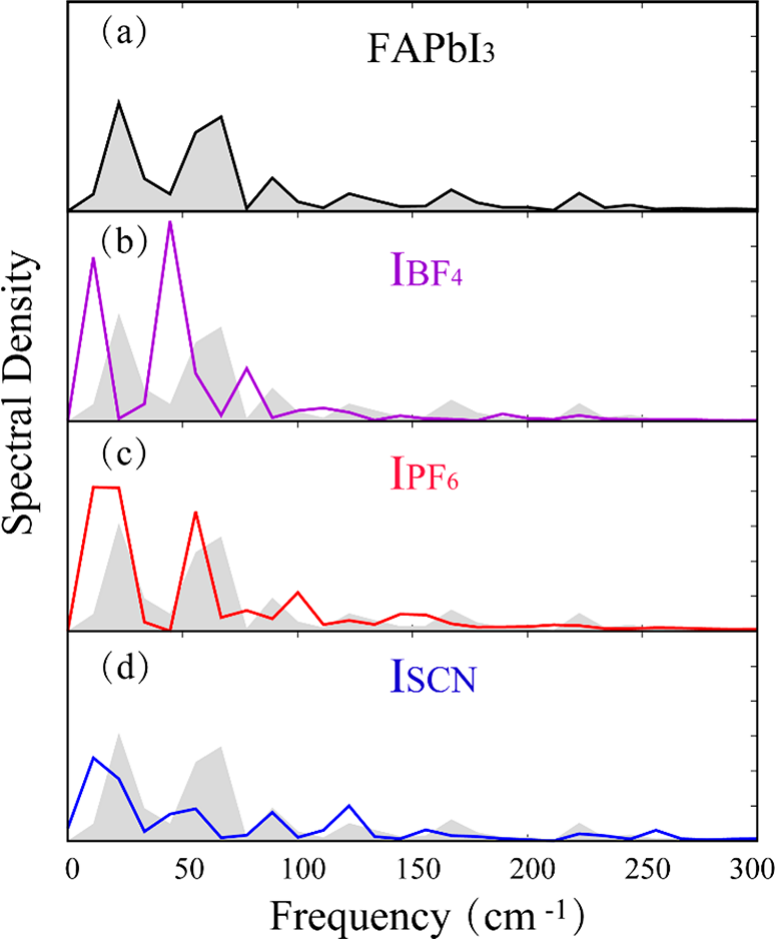
Spectral densities obtained by Fourier transforms of fluctuations of the electron–hole energy gaps in FAPbI_3_ (a), I_BF_4__ (b), I_PF_6__(c), and I_SCN_ (d).

Figure 5 presents the pure dephasing functions *D*(*t*) for electron–hole recombination in FAPbI_3_, I_BF_4__, I_PF_6__, and I_SCN_, which were computed using the second-order cumulant approximation of the optical response theory:^35^
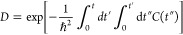
4Here, *C*(*t*) is the unnormalized ACF (eq 2). Fitting the functions to a Gaussian, exp[−0.5(*t/τ*)]^2^ gives the pure dephasing times τ, as reported in Table 2. The short 10 fs coherence times contribute to the long-lived excited-state lifetime in perovskites, as exemplified by the quantum Zeno effect,^74^ according to which quantum dynamics stops in the limit of infinitely fast decoherence. The coherence times are short because the electrons and holes are weakly correlated. They are localized on different atoms, Pb and I, and couple to a broad range of anharmonic^76,77^ vibrations (Figure 4). The disorder introduced by the pseudohalide dopants slightly shortens the coherence times. The pure dephasing times can be correlated to the ACF initial values (insert in Figure 5), which are equal to the square root of the electronic bandgap fluctuation. Generally, greater unnormalized ACF initial values lead to faster pure dephasing times.^35^ Thus, the initial ACF value is the largest for I_BF_4__, and the corresponding pure dephasing time is the shortest. The pure dephasing time is the longest for pristine FAPbI_3_, but the corresponding initial ACF value is not the smallest. Coherence is the longest for pristine FAPbI_3_ because the ACF shows the most regular oscillation among the four systems, and the positive and negative ACF contributions to the integral in eq 4 cancel the most.

**Figure 5:**
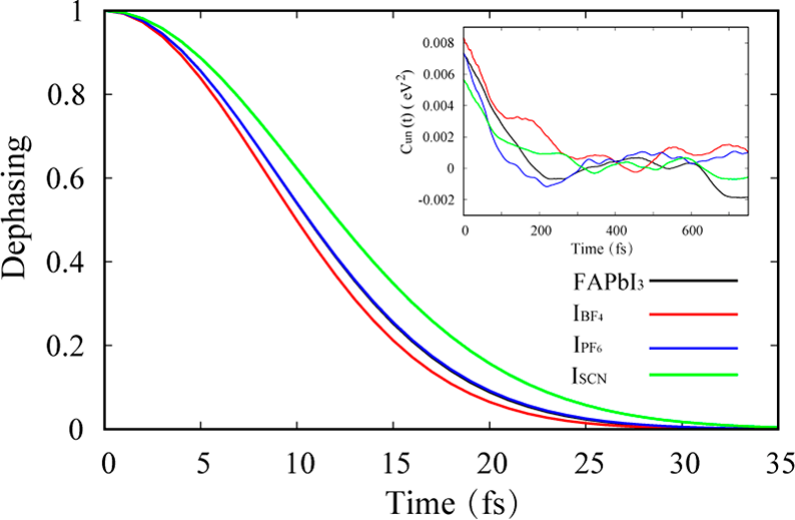
Pure dephasing functions for electron–hole recombination in FAPbI_3_, I_BF_4__, I_PF_6__, and I_SCN_. The inset shows the unnormalized autocorrelation functions, whose initial values are the bandgap fluctuations squared.

Nonradiative charge carrier recombination depends primarily on the NAC, which is governed by the relative localization of the initial and final (electron and hole) wave functions, the energy gap, and the atomic velocity (eq 1). The pure dephasing times also influence the recombination; however, they were very similar (Table 2). The bandgaps were similar as well. All of the systems were considered at the same temperature, and therefore, the atomic velocity was smaller for lower-frequency motions (Figure 4). The overlap of the electron and hole wave functions depends on structural disorder. The pseudohalide doping increased the disorder and shifted the spectral density to lower frequencies, which both contributed to the observed longer recombination times. The evolution of the excited-state populations during the nonradiative electron–hole recombination dynamics are shown in Figure 6. The nonradiative decay times, reported in Table 2, were obtained by fitting the data to the short-time linear approximation of the exponential decay: *P*(*t*) = exp(−*t*/τ) ≈ 1 – *t*/τ. The results confirm the expectation based on the above analysis. All of the pseudohalides slowed the charge carrier recombination, with the longest lifetime observed for PF_6_^–^. Its lifetime increased by a factor of 2.5 relative to that of pristine FAPbI_3_. PF_6_^–^ created the largest local lattice distortion (Table 1), breaking the lattice symmetry and reducing the electron–hole overlap the most. The overall recombination was slow because the NA coupling was small (4 meV) and the pure dephasing time was short (10 fs).

**Figure 6:**
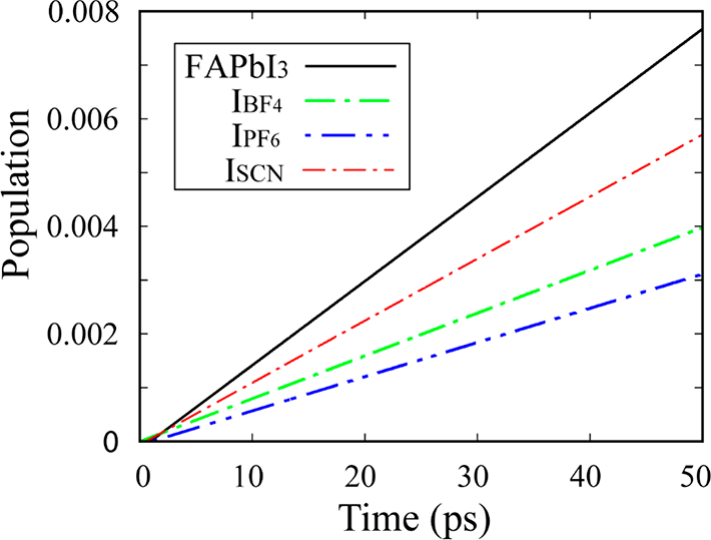
Electron–hole recombination dynamics in FAPbI_3_, I_BF_4__, I_PF_6__, and I_SCN_.

In conclusion, we performed NAMD simulations combined with real-time TD-DFT to investigate nonradiative charge carrier recombination in FAPbI_3_ doped with three pseudohalide anions: SCN^–^, BF_4_^–^, and PF_6_^–^. Using these anions as a substitute for an iodide in FAPbI_3_ created a local structural distortion and shifted the relevant vibrations to lower frequencies. Consequently, the electrons and holes became partially localized and overlapped less, while at the same time, the atomic motions driving the nonradiative charge recombination became slower. Both of these factors reduced the NAC responsible for the recombination, and the excited-state lifetime became longer. The lifetime correlated directly with the pseudohalide-induced local structural distortion and increased in the order of FAPbI_3_ < SCN^–^ < BF_4_^–^ < PF_6_^–^. The bandgap and the pure dephasing times, which also influence the charge carrier lifetime, changed little upon pseudohalide doping. Overall, the nonradiative processes were slow because they were driven by low-frequency anharmonic motions of the heavy Pb–I inorganic lattice and because the electrons and holes were localized on different widely spaced atoms, Pb and I, and overlapped little. The NAC coupling was weak, being only a few meV, and the quantum coherence was short (10 fs); both of these factors favor long lifetimes. The low-frequency anharmonic motions created dynamic disorder, which was enhanced by the dopants, further decreasing the electron–hole overlap. The atomistic analysis of the mechanisms of charge and energy losses in metal halide perovskites provided by the current study contributes to the fundamental understanding of perovskite properties, which is needed for the rational design of efficient solar energy and optoelectronic materials.
